# Glycoside hydrolases family 20 (GH20) represent putative virulence factors that are shared by animal pathogenic oomycetes, but are absent in phytopathogens

**DOI:** 10.1186/s12866-016-0856-7

**Published:** 2016-10-06

**Authors:** Isabel E. Olivera, Katrina C. Fins, Sara A. Rodriguez, Sumayyah K. Abiff, Jaime L. Tartar, Aurélien Tartar

**Affiliations:** 1 Department of Biological Sciences, Nova Southeastern University, Fort Lauderdale, FL USA; 2 Department of Psychology and Neuroscience, Nova Southeastern University, Fort Lauderdale, FL USA

**Keywords:** Crinkler, CRN, Effector, *Lagenidium giganteum*, Entomopathogen, Hexosaminidase

## Abstract

**Background:**

Although interest in animal pathogenic oomycetes is increasing, the molecular basis mediating oomycete-animal relationships remains virtually unknown. Crinkler (CRN) genes, which have been traditionally associated with the cytotoxic activity displayed by plant pathogenic oomycetes, were recently detected in transcriptome sequences from the entomopathogenic oomycete *Lagenidium giganteum*, suggesting that these genes may represent virulence factors conserved in both animal and plant pathogenic oomycetes. In order to further characterize the *L. giganteum* pathogenome, an on-going genomic survey was mined to reveal novel putative virulence factors, including canonical oomycete effectors Crinkler 13 (CRN13) orthologs. These novel sequences provided a basis to initiate gene expression analyses and determine if the proposed *L. giganteum* virulence factors are differentially expressed in the presence of mosquito larvae (*Aedes aegypti*).

**Results:**

Sequence analyses revealed that *L. giganteum* express CRN13 transcripts. The predicted proteins, like other *L. giganteum* CRNs, contained a conserved LYLA motif at the N terminal, but did not display signal peptides. In contrast, other potential virulence factors, such as Glycoside Hydrolases family 20 (hexosaminidase) and 37 (trehalase) proteins (GH20 and GH37), contained identifiable signal peptides. Genome mining demonstrated that GH20 genes are absent from phytopathogenic oomycete genomes, and that the *L. giganteum* GH20 sequence is the only reported peronosporalean GH20 gene. All other oomycete GH20 homologs were retrieved from animal pathogenic, saprolegnialean genomes. Furthermore, phylogenetic analyses demonstrated that saprolegnialean and peronosporalean GH20 protein sequences clustered in unrelated clades. The saprolegnialean GH20 sequences appeared as a strongly supported, monophyletic group nested within an arthropod-specific clade, suggesting that this gene was acquired via a lateral gene transfer event from an insect or crustacean genome. In contrast, the *L. giganteum* GH20 protein sequence appeared as a sister taxon to a plant-specific clade that included exochitinases with demonstrated insecticidal activities. Finally, gene expression analyses demonstrated that the *L. giganteum* GH20 gene expression level is significantly modulated in the presence of mosquito larvae. In agreement with the protein secretion predictions, CRN transcripts did not show any differential expression.

**Conclusions:**

These results identified GH20 enzymes, and not CRNs, as potential pathogenicity factors shared by all animal pathogenic oomycetes.

**Electronic supplementary material:**

The online version of this article (doi:10.1186/s12866-016-0856-7) contains supplementary material, which is available to authorized users.

## Background

Oomycetes are fungal-like heterokonts that are principally known as plant pathogens [1]. The most extensively studied oomycete genus, *Phytophthora*, includes the Irish potato famine pathogen, *P. infestans*, and remains responsible for serious economic losses to crops worldwide [1]. Although over 60 % of all described oomycetes are recognized as plant pathogens, recent evidence has suggested that oomycetes evolved from a marine animal pathogen [2], and that phytopathogenicity was acquired independently as a derived, apomorphic trait in multiple oomycete clades [3]. The transition to plant pathogenicity has been associated with a dramatic expansion of the Crinkler (CRN) gene family in the genomes of phylogenetically distant phytopathogenic oomycetes, such as the Peronosporalean *P. infestans* and the Saprolegnialean *Aphanomyces euteiches* [4]. In accordance with this hypothesis, CRN genes were shown to be absent from genome sequences generated from basal (non-plant pathogen) oomycetes [5]. Canonical CRN effector proteins are characterized by the conserved LxLYLA or LxLFLA motifs at the N terminal, which have been implicated in the transport of these proteins in the host cells during plant infection. Following translocation in the host cells, CRN proteins accumulate in the nucleus, where they induce cell death [4].

The entomopathogen *Lagenidium giganteum* represents a unique, extant animal pathogenic oomycete that has been shown to express canonical CRN oomycete effector genes [6]. This observation, combined with phylogenetic analyses that placed *L. giganteum* nested within a Peronosporalean clade of plant pathogens, suggested that *L. giganteum* evolved from a plant pathogenic ancestor, and may have reverted back towards a plesiomorphic-like state. The presence of CRN sequences in the *L. giganteum* transcriptome contrasted with historic observations describing this oomycete as a mosquito pathogen with a narrow range of invertebrate hosts, and little impact on plant tissues [6]. Interestingly, the true nature of the *L. giganteum* host range has also been recently challenged by reports of *L. giganteum* infections in mammals [7]. These reports have diminished the potential of *L. giganteum* as a biocontrol agent against mosquitoes. However, they also have contributed to reinforce the original assertions that *L. giganteum* is defined as a mosquito pathogen, since this characteristic phenotypical feature was used to complement molecular-based phylogenetic analyses, and validate the identification of mammalian pathogenic *Lagenidium* spp. as *L. giganteum* [7]. Both historic [8] and recent [7] isolations of *L. giganteum* have demonstrated that one of the most characteristic attributes of this organism is its ability to infect and kill mosquito larvae, legitimating the hypothesis that the *L. giganteum* genome represents a valuable source of novel bioactive compounds with potential as bioinsecticides against mosquitoes [6].

An alternative hypothesis that reconciles the presence of CRN genes in the *L. giganteum* transcriptome and its pathogenicity to animals, proposes that CRN proteins may play a role during mosquito infection [6]. This hypothesis is mainly supported by the fact that CRN genes have been detected in the genome of the chytrid fungus *Batrachochytrium dendrobatidis* (Bd), which is predominantly known as a devastating frog pathogen that threatens natural amphibian populations worldwide [9]. Differential gene expression analyses indicated that *BdCRN* genes were up-regulated in the presence of frog skin [9], suggesting that CRN proteins may represent pathogenicity factors that are also active on animal cells. Parallel investigations of CRN proteins originating from Bd and the plant pathogen oomycete *Aphanomyces euteiches* have focused on one specific CRN sequence, known as CRN13 [10], and have demonstrated that homologous proteins of different origins shared similar functions at the molecular level (DNA binding), concentrate to similar location in the respective host cells (nucleus), and lead to similar outcomes (host cell death). The *L. giganteum* CRN proteins have yet to be included in such comparative analyses, and it remains unclear if they include a CRN13 homolog, and if they have any role in the pathogenicity process. Overall, the molecular processes mediating mosquito infections by *L. giganteum* remain uncharacterized. A recent study reported that *L. giganteum* secretes GH5_27 enzymes that appear to be mostly specific to cuticle degrading entomopathogens, including not only entomopathogenic oomycetes but also Fungi [6]. Earlier reports have proposed trehalases as potential pathogenicity factors, based on the observations that trehalose is the most abundant sugar source in insects’ hemolymph, and that trehalose depletion may contribute to the *L. giganteum* infection process and ultimate death of the insect host [11].

In this study, an on-going survey of the *L. giganteum* genome and transcriptome was used to characterize the *L. giganteum* gene sequences for CRN13 and trehalase (Glycoside Hydrolase family 37, or GH37) orthologs. The CRN13 and GH37 nucleotide sequences provided a basis to initiate differential gene expression studies, and estimate if these genes are up-regulated in the presence of *Aedes aegypti* mosquito larvae, which are demonstrated natural hosts for *L. giganteum* [12]. The differential expression studies were performed in an effort to develop comparative analyses between Bd and *L. giganteum*, and establish if CRN13 proteins represent virulence factors shared by unrelated animal pathogens. The gene expression analyses presented herein also included the previously reported *L. giganteum* oomycete effector genes (elicitin, CRN and Cellulose Binding Elicitor Lectin, or CBEL, orthologs), as well as the entomopathogen-specific GH5_27 gene [6]. Finally, a novel *L. giganteum* Glycoside Hydrolase family 20 (GH20) enzyme is reported in this study. Several GH20 gene fragments were detected in the on-going *L. giganteum* transcriptome survey using a previously published screening rationale [6]. The fragment sequences exhibited little similarity with publicly available oomycete sequences. Therefore, the complete coding region was generated and the GH20 gene was incorporated in the gene expression analyses. Overall, this study provides insight into the gene expression profiling of an oomycete principally known as an animal pathogen. It anticipates the upcoming release and analysis of the *L. giganteum* genome sequence, and contributes to the development of workflows aimed at the identification and functional characterization of virulence factors with potential biological activities against mosquitoes.

## Results

### Sequence analysis of *L. giganteum* CRN13 homologs

The use of the CRN13 primers in internal and RACE PCRs revealed two different CRN13 homologs for *L. giganteum* (not shown). The complete sequences of these two transcripts were 1341 bp and 1260 bp long, respectively, and were deposited in the GenBank/EMBL/DDBJ databases under the accession numbers KX269151 and KX269152. Homology searches demonstrated that the C terminals of both predicted protein sequences contained the DFA and DDC putative oomycete effector subdomains (based on the nomenclature established for the *Phytophthora infestans* CRN proteins [13]). These subdomains were recently further characterized in the oomycete *Aphanomyces euteiches* and the chytrid fungus *Batrachochytrium dendrobatidis* [10]. The HNH-like motif that was identified in CRN13 proteins and associated with host DNA binding properties [10] was also conserved in *L. giganteum*, and located within the DFA subdomains. Only one of the two *L. giganteum* sequences (GenBank accession number KX269151) displayed the canonical LxLYLAR/K and HVLVxxP motifs that are characteristic of the N terminal regions of oomycete CRN genes. These domains have been implicated with protein transfer into the host plant cells [4], and, in agreement with these observations, the CRN homologs of the animal pathogen *B. dendrobatidis* have been mostly associated with two distinct N terminal motifs referred to as type A and type B [14]. Homology searches aimed at characterizing the non-canonical *L. giganteum* protein (GenBank accession number KX269152) did not reveal any similarity between the *L. giganteum* and *B. dendrobatidis* CRN13 N-terminals, and indicated that both *L. giganteum* proteins, including N terminals, showed stronger identities to CRN or CRN-like proteins reported from *Phytopththora* spp. (not shown). Sequence analyses demonstrated that the *L. giganteum* CRN13 homologs did not contain identifiable signal peptides, mirroring previous observations reported for other *L. giganteum* CRN homologs [6]. Further analyses indicated that the *L. giganteum* CRN13 protein sequences were not associated with non-classical secretory pathways, with NN scores of 0.33 and 0.29 (below the threshold of 0.5) [15].

### Characterization of the *L. giganteum* GH20 and GH37 transcripts

In contrast to the CRN13 proteins, signal peptides were predicted for the *L. giganteum* GH20 and GH37 homologs. RACE PCRs produced complete transcript sequences that were 1973 bp and 1968 bp-long for GH20 and GH37, respectively. The sequences were deposited in the GenBank/EMBL/DDBJ databases under the accession numbers KX269153 (GH20) and KX269154 (GH37). Motif searches using InterProScan demonstrated that the signal peptides preceded a single Glycoside Hydrolase (GH) domain for both predicted proteins. The domains were further identified as GH family 20 (GH20, IPR025705) or GH family 37 (GH37, IPR001661), which have been associated with β-hexosaminidase (EC 3.2.1.52) and trehalase (EC 3.2.1.28) activities, respectively. In addition, GH20 enzymes have also been linked to chitinase activity, and exoskeleton degrading processes in insects and crustaceans [16]. Homology searches and genome mining demonstrated that trehalase genes were widespread in oomycetes, as previously reported [5], and indicated that the *L. giganteum* GH37 protein sequence shared 56 % identity with homologous proteins from the plant pathogens *Phytophthora infestans* and *P. nicotianae*, and 49 % sequence identity with trehalase proteins from the fish pathogen *Saprolegnia parasitica*. In contrast, GH20 genes appeared completely absent from plant pathogens, and have been identified only in a small sample of animal pathogenic oomycetes, including *S. parasitica* and the decapod parasite *Achlya hypogyna* [5]. Genome mining using the FungiDB database confirmed that oomycete GH20 orthologs have only been reported in the Saprolegnialeans, and revealed that these genes are virtually restricted to animal pathogens. A total of six GH20 protein sequences were retrieved from public databases, and these sequences originated from the fish pathogens *S. parasitica* (XP_012206853), *S. diclina* (XP_008611584) and *Aphanomyces invadans* (XP_008874997), the crayfish pathogen *A. astaci* (XP_009833685), the decapod parasite *Ac. hypogyna* (AIG55828) and the free living *Thraustotheca clavata* (AIG55611). Although all 6 Saprolegnialean sequences shared significant similarity (Additional file 1), preliminary sequence comparisons performed through homology searches indicated that the predicted *L. giganteum* GH20 protein sequence was more similar to plant sequences than all other oomycete sequences, and prompted more comprehensive phylogenetic analyses.

### GH20 phylogenetic analysis

**Fig. 1:**
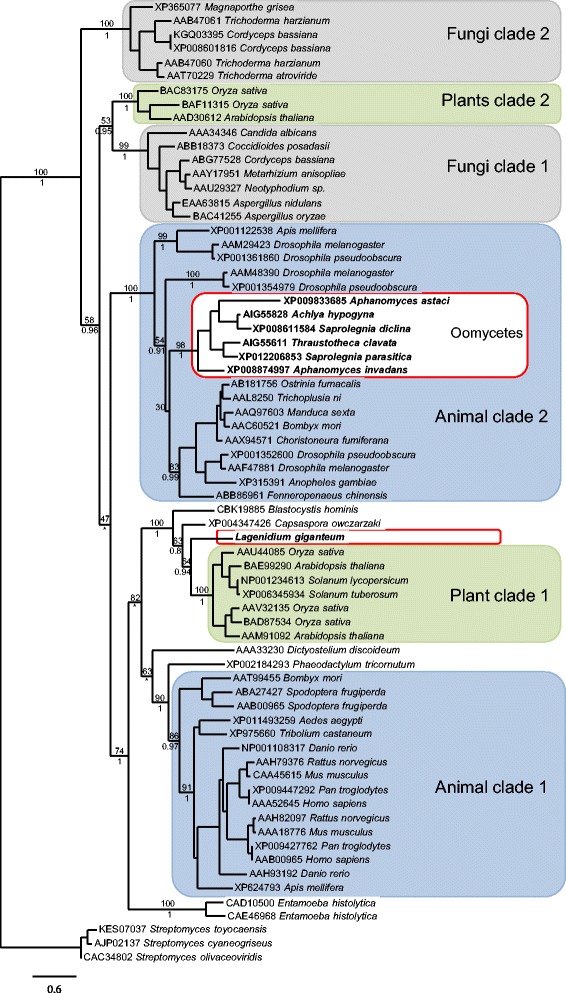
Maximum Likelihood (ML) phylogram inferred from eukaryotic Glycoside Hydrolase family 20 (GH20) amino acid sequences (443 characters). The tree is consistent with previously published GH20 phylogeny reconstructions, and shows that oomycete GH20 proteins (in *bold*, and *circled in red*) cluster in two distinct groups. The Saprolegnialean sequences appear as a strongly supported cluster nested within an arthropod-specific clade (animal clade 2, in *blue*). In contrast, the *L. giganteum* sequence appears as a sister taxon to a plant clade (plant clade 1, in green). All but one sequences were retrieved from animal pathogenic oomycete genomes, as GH20 were shown to be absent from plant pathogenic oomycetes. Numbers above the modes correspond to ML bootstrap values (1000 replicates). Numbers below the nodes correspond to Bayesian posterior probabilities (shown only when above 0.5). Asterisks indicate changes in topology between trees inferred from ML or Bayesian analyses. For clarity purposes, not all values representative of the support for nodes within each major clade are shown. The bar indicates the number of substitutions per site

The phylogram inferred from the Maximum Likehood (ML) analyses is presented in Fig. 1. The phylogenetic tree was rooted with bacterial GH20 proteins, and its topology was very consistent with the consensus tree obtained from Bayesian analyses (Fig. 1), confirming observations that ML and Bayesian analyses correlate with one another, generally outperforming other methods such as maximum parsimony [17]. In addition, both trees were congruent with previously published eukaryotic GH20 protein phylogeny reconstructions inferred from ML and Bayesian analyses [16]. Especially, the trees depicted very strongly supported clades corresponding to multiple paralogous subfamilies of fungal, plant or animal GH20 enzymes (Fig. 1). Sequences from each of these groups are split between pairs of monophyletic, strongly supported clusters (Fig. 1), suggesting a potential gene duplication event in the common ancestor of plants, animals and fungi [16]. Fungal and plant clusters are respectively labeled as fungal clades 1 and 2, and plant clades 1 and 2 (Fig. 1), following a previously proposed nomenclature [16]. Similarly, animal sequences are divided into two clusters that include the vertebrate GH20 alpha and beta chains as sister clades (animal clade 1), and an arthropod-specific GH20 clade (animal clade 2). Deeper nodes, indicative of the relationships between the different clades, were characterized by weak statistical support in both current (this study) and previous [16] analyses. However, similar patterns emerging from both studies included the observations that fungal clade 1 and plant clade 2 clustered as sister clades, and that animal clade 1 and plant clade 1 appeared more closely related to each other than any other clades (Fig. 1).

In all analyses, the phylogenetic trees depicted the *L. giganteum* GH20 protein as a sister taxon to the plant clade 1 (Fig. 1). Plant clade 1 includes proteins with known exochitinase [18] and insecticidal activities [19]. In particular, corn tissues genetically modified to express the *Arabidopsis thaliana* GH20 BAE99290 transcript caused mortality or reduced growth rate of pest insects [19], suggesting that the *L. giganteum* GH20 proteins could have similar effects on the mosquito hosts. The *L. giganteum* GH20 sequence also appeared as a close relative to other heterokont, but non-oomycete, sequences from *Blastocystis hominis* and *Phaeodactylum tricornutum* (Fig. 1). These relationships suggest that the *L. giganteum* homolog represents an ancestral gene sequence. Although they appear as a paraphyletic assemblage, and not a monophyletic group (Fig. 1), the position of heterokont sequences (*L. giganteum*, *B. hominis*) as close relatives to plant sequences is consistent with recent reconstructions of eukaryote phylogeny [20, 21], and supports the hypothesis that the *L. giganteum* GH20 gene was acquired vertically.

The oomycete GH20 sequences from Saprolegnialeans were not associated to any other heterokont sequences (Fig. 1). They appeared as a strongly supported monophyletic clade, and this clade was nested within the arthropod-specific animal clade 2 (Fig. 1). This topology suggests that the Saprolegnialean GH20 gene was acquired from a lateral gene transfer event from an arthropod to the Saprolegnialean ancestor. Although a recent study focused on oomycete ancestral secretome identified multiple candidate genes acquired by oomycetes via horizontal gene transfer, the GH20 gene was not included in this report [5]. The phylogenetic analysis presented in our study (Fig. 1) provides a basis to initiate further phylogeny reconstructions to support the hypothesis of an animal to oomycete gene transfer. In particular, the addition of non-insect, invertebrate sequences may complement the single *Fenneropenaeus chinensis* (ABB86961) sequence, and provide improved resolution for the nodes that appear only moderately supported by statistical analyses (Fig. 1). Since some of the sampled Saprolegnialean oomycetes (*A. astaci* and *Ac. hypogyna*) are known pathogens of crustaceans, a testable hypothesis of refined phylogeny reconstructions may focus on a host-to-pathogen lateral gene transfer, confirming previous reports that such transfers are important events contributing to the evolution of pathogenic traits [22, 23].

Even if the origin of these genes remains to be comprehensively determined, genome mining demonstrated that GH20 genes are shared by all animal pathogenic oomycetes (including *L. giganteum*) and are absent from plant pathogen genomes. The GH20 proteins were predicted to be secreted by all animal pathogens. In addition, phylogenetic analyses strongly suggested that the *L. giganteum* GH20 proteins may display chitinase and insecticidal activities. In order to provide further evidence that the GH20 proteins have a role in mosquito infection, the gene expression pattern of the *L. giganteum* GH20 gene was investigated.

### Differential gene expression analysis

**Fig. 2:**
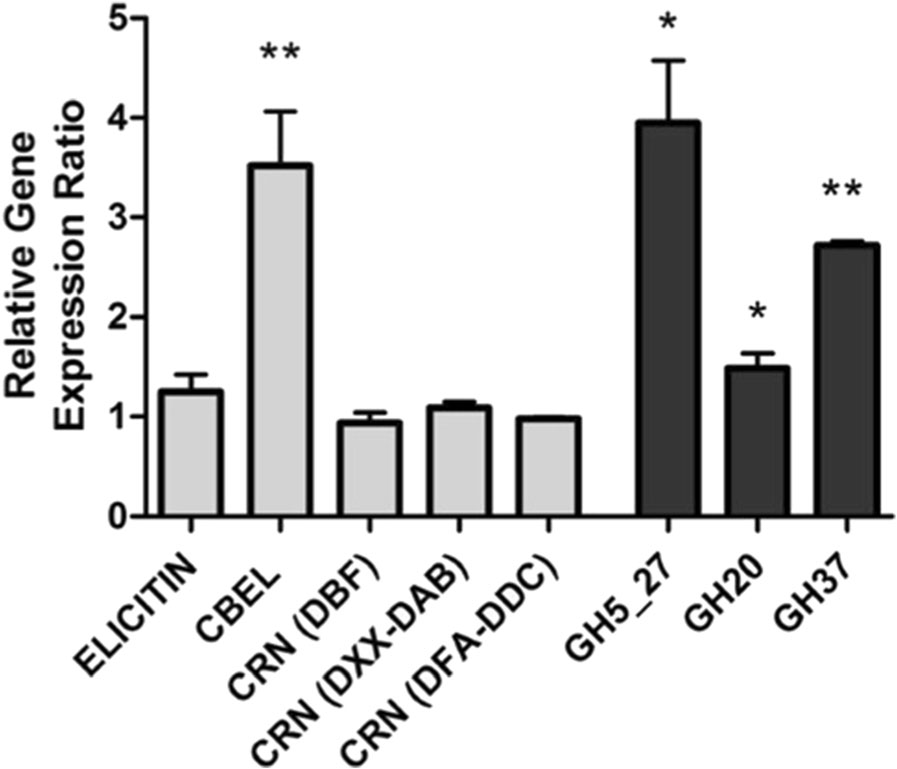
Ratios of relative expression levels of selected *L. giganteum* gene transcripts in presence vs. absence of mosquito larvae (*Aedes aegypti*). Canonical oomycete effectors, characteristic of plant pathogenic oomycetes, are color coded in light grey (left), whereas carbohydrate active enzymes, including the phylogenetically unique *L. giganteum* GH5_27 and GH20, are represented in dark grey (right). Relative expression levels for each gene (*n* = 4) were normalized to the control genes β-tubulin and cellulose synthase. Error bars represent standard error of the mean. * indicates *p* < 0.05, ** indicates *p* < 0.01

The differential expression analyses were preceded by the identification of a *L. giganteum* intron in a conserved location of the 5’ end of the cellulose synthetase 3 gene [24]. This 76 bp long intron was amplified and sequenced from genomic DNA preparations, and the sequence was deposited in the GenBank/EMBL/DDBJ databases under the accession number KX269155. Importantly, the presence of a conserved intron allowed for control reactions (not shown) that confirmed the absence of gDNA contamination in all the *L. giganteum* cDNA preparations that were used to determine gene expression profiles in the absence vs. presence of mosquito hosts. The gene expression analyses are presented in Fig. 2. These analyses revealed that four out of the eight tested *L. giganteum* genes were significantly differentially expressed, and showed increased expression, in the presence of mosquito larvae (Fig. 2). Independent samples t tests indicated that, relative to a ratio of 1, gene expression was significantly increased for all the Glycoside Hydrolases proposed as virulence factors to date, including the invertebrate specific GH5_27 (mean = 3.13, SD = 1.4), t(6) = -3.04, *p* < 0.05, the newly identified GH20 (mean = 1.49, SD = 0.30), t(6) = -3.57, *p* < 0.05, and GH37 (mean = 2.72, SD = 0.08), t(6) = -3.57, *p* < 0.001. Unlike the GH genes, most phytopathogenic-like effectors, including elicitin and CRN genes, do not show any differential expression (Fig. 2). Transcripts corresponding to the Cellulose Binding Elicitor Lectin (CBEL) proteins, which have been previously associated with the *L. giganteum* attachment to carbohydrate residues potentially embedded in the chitin-based host cuticle [6], were the only canonical oomycete effectors [25] to appear significantly up regulated in the presence of host substrate (mean = 0.29, SD = 0.21), t(6) = 69.94, *p* < 0.001. Importantly, the gene expression analysis (Fig. 2) complemented the protein secretion predictions that were based on the identification of signal peptide at the N terminal of the putative *L. giganteum* proteins (above). The genomic sequence information collected in the current and previous [6] studies has indicated that the *L. giganteum* CRN proteins appear to lack signal peptide sequences, and are not overexpressed in the presence of the host. In contrast, all genes that were demonstrated to be significantly up-regulated in the presence of mosquito larvae (Fig. 2) were also characterized by the presence of signal peptides, suggesting that they represent promising virulence factor against mosquitoes.

## Discussion

One of the main objectives of this study consisted of determining if the *L. giganteum* transcriptome contains CRN13 orthologs, and if these genes are over-expressed in the presence of (mosquito) host substrates. The genomic survey revealed that CRN13 orthologs, including sequences characterized by the canonical association of the LxLYLA-DFA-DDC motifs [10], are present in the *L. giganteum* genome, and the fact that these genes can be readily amplified from RNA pools suggested that the CRN13 proteins are expressed by *L. giganteum* cells. The presence of CRN13 homologs provides evidence to support the recent description of CRN transcripts in the *L. giganteum* transcriptome [6], expanding the understanding of both the *L. giganteum* effector repertoire, and its phylogenetic affinities. However, the characterization of *L. giganteum* CRN putative proteins also indicated that these effector orthologs may play little role in the mosquito pathogenicity process. First, all CRN protein sequences obtained to date were characterized by the absence of signal peptides, suggesting that the corresponding proteins are not secreted during host-pathogen interactions. In addition, none of the three *L. giganteum* CRN canonical C terminal domains (DBF, DXX-DAB, and DFA-DDC) appeared up regulated in the presence of host substrate (Fig. 2). The lack of both detectable signal peptides and up-regulation during host interactions contrast with the situation reported for the frog pathogen *B. dendrobatidis*. In Bd, at least some of the putative CRN proteins were predicted to contain signal peptides [14], and showed significant increased expression in the presence of frog skin [9]. For *L. giganteum*, the evidence generated to date has remained consistently insufficient to link CRN proteins to the pathogenicity process. A comprehensive analysis of the *L. giganteum* CRN effector repertoire may require not only the complete genome sequence for this oomycete, but also an improved sampling of mosquito infection time points. As noted previously, *L. giganteum* causes systemic infections in host insects [6]. The infectious process is similar to other filamentous entomopathogens, such as the fungus *Beauveria bassiana*, and can be divided into distinct, sequential steps that may be associated with specific molecular arsenals [26]. These steps include the recognition and attachment of zoospores to the host cuticle, followed by zoospore germination and penetration of a germ tube through the exoskeleton, and finally evasion of the host defense system, and mycelial growth, within the host hemolymph. The gene expression analyses presented in this study were designed to initiate comparisons with Bd, and mimic the addition of a basic host substrate (frog skin) by supplementing the growth media with mosquito larvae. Accordingly, the putative virulence factors identified through these analyses may be expected to exhibit biological activity on the insect cuticle, and predominantly play a role in the early stage of infection (cuticle attachment and penetration). This initial profiling may need to be complemented with additional gene expression studies aimed at identifying *L. giganteum* proteins mediating the *in vivo* interactions with the host defense system during later infection stages. It is possible that the complete genome sequence of *L. giganteum* will reveal CRN proteins amended with predicted signal peptides, and that comprehensive transcriptomics and proteomics analyses of the *in vivo* host-pathogen interactions will indicate that CRN proteins are secreted in the insect hemolymph and contribute to the pathogenicity process. However, the global current evidence indicates that CRN proteins are not involved in entomopathogenic interactions, supporting the hypothesis that these effectors may represent remnant characteristics of the *L. giganteum* phytopathogenic ancestor [6].

Although the *L. giganteum* secretome appears devoid of CRN proteins, it includes a unique combination of carbohydrate-active molecules, containing either Carbohydrate-Binging Module (CBM1) or Glycoside Hydrolases (GH) domains, that have been characterized not only by the presence of predicted signal peptides, but also by high levels of gene expression in the presence of mosquito larvae (Fig. 2). This growing *L. giganteum* CAZome [27] represents a promising catalog of virulence factors that may exhibit significant biological activity against *A. aegypti*, which is known as the predominant mosquito vector for numerous current public health threats, including dengue, chikungunya and zika fevers [28]. In particular, the strong up-regulation of the CBEL gene (Fig. 2) supports the hypothesis that these proteins mediate the *L. giganteum* attachment to the mosquito host cuticle [6], and suggests that the CBM1 domains may have important biotechnological applications for the development of novel contact bioinsecticides. The CBM1 domains have also been detected in the genome sequences of entomopathogenic fungi, sometimes in association with GH18 (chitinase) motifs [29]. Since the CBM1 domains are predominantly linked with binding to cellulose, they have been seldom related to the pathogenicity process in entomopathogenic fungi, and instead have been tied to the endophytic abilities displayed by many fungal entomopathogens [30]. However, fungal CBM1 domains can also bind to chitin, and improved the substrate binding and activity of chimeric chitinases [31], leading to increased pathogen virulence on chitin-based host substrate [32]. Similarly, fungal GH37 enzymes have been implicated in the early stages of insect infection by the filamentous entomopathogen *Metarhizium anisopliae* [33]. These reports contribute to validate the gene expression assays performed in this study and provide additional evidence that the up-regulated *L. giganteum* CAZome illustrated in Fig. 2 includes important pathogenicity determinants. They also confirm the previously proposed hypothesis that entomopathogenic filamentous pathogens (fungi and oomycetes) exhibit convergent evolution [6]. Furthermore, the fact that at least some of the *L. giganteum* putative pathogenicity factors, such as CBEL and GH37, are significantly up-regulated (*p* < 0.01) in the presence of mosquito larvae serves to strengthen the observation that the CRN proteins may not be involved in the infectious process.

Finally, the gene expression analyses revealed that two novel, and phylogenetically unique, *L. giganteum* genes (GH20 and GH5_27) are differentially expressed in the presence of host substrates (Fig. 2). The GH5_27 predicted proteins were originally described in a previous transcriptome study [6]. They were shown to be absent from most plant pathogenic genomes, but shared by phylogenetically diverse cuticle-degrading organisms, including insect and nematode pathogens. The robust change in gene expression demonstrated in this study confirms that the GH5_27 enzymes represent promising compounds that warrant being tested for insecticidal potential (cuticle degradation). Analogously, genome mining and phylogenetic analyses performed in this study (Fig. 1) established that GH20 enzymes are absent in phytopathogens, but are shared by animal pathogenic oomycetes. Although the change in expression appeared subtler, statistical analyses showed that the GH20 transcripts are included in the significantly up-regulated *L. giganteum* secretome (Fig. 2). The significance of the identification of the *L. giganteum* GH20 gene is two-fold. On one hand, the detection of molecules with strong similarity to known chitinases suggests that the *L. giganteum* GH20 enzymes should be included along with the GH5_27 proteins in functional studies aimed at investigating the impact of these molecules on the host cuticle. The potential of the GH20 enzymes as mosquitocides should also be evaluated, based on combined reports describing hexosaminidases as insecticidal toxins [19], and *L. giganteum* extracellular metabolites as larvicidal compounds [34]. On the other hand, the arthropod-to-oomycete lateral gene transfer proposed for Saprolegnialean GH20 genes not only supports the hypothesis that GH20 enzymes are important pathogenicity factors for *L. giganteum*, but also indicates that a wider sampling of animal pathogenic oomycete genomes may lead to the identification of shared pathogenicity factors active on animal hosts. This shared set of genes appears to include GH20 enzymes, but not CRN proteins, and may be contrasted to the common core of effectors that was identified from the wealth of genomic information produced for plant pathogenic oomycetes [25]. Studies have been initiated to confirm the presence of GH20 genes in other, previously unsampled, animal pathogenic oomycetes, such as the mosquito pathogen *Leptolegnia chapmanii*, or the nematode pathogen *Lagenidium caudatum* [6]. The generation of additional sequences will serve to refine the phylogenetic analyses presented in Fig. 1, in an effort to resolve the relationships between the various fungal, animal, and plant clades, and provide support for a host-to-pathogen lateral gene transfer event [22, 23] in the Saprolegnialean ancestor.

## Conclusions

The gene expression analyses presented in this study contribute to the identification of several *L. giganteum* genes that are up-regulated in the presence of mosquito host, and complement the gene sequence annotation initiated by a *L. giganteum* transcriptome survey [6]. The most promising virulence factor candidates correspond to proteins predicted to be active on host cuticle carbohydrates, and include GH20 enzymes that may represent novel pathogenicity factors shared among animal pathogenic oomycetes. Functional studies aimed at demonstrating the predicted activities of GH20, GH5_27, GH37 and CBEL proteins on mosquito larvae are currently being initiated by cloning the reported full-length gene sequences in expression vectors. The purification of recombinant proteins will allow for the production of antibodies that may be used in immunoblotting reactions to confirm secretion predictions, and gene expression patterns, at a proteomics level. Overall, the gene profile information presented in this study provides an additional line of evidence to validate the entomopathogen *L. giganteum* as a source of novel biological compounds against vector mosquitoes, and as a strong model to uncover the fundamental molecular mechanisms underlying pathogenicity in animal pathogenic oomycetes [35].

## Methods

### Microbial culture and DNA/RNA extraction

The oomycete *Lagenidium giganteum* (ARSEF #373) was obtained and maintained in axenic cultures as previously described [6]. Genomic DNA (gDNA) and total RNA were extracted from liquid cultures using the QIAGEN DNeasy or RNeasy Plant minikits, respectively [6].

### Amplification and sequencing of *L. giganteum* CRN13 orthologs

Fragments corresponding to CRN13 orthologs were directly amplified from *L. giganteum* cDNA using the primers CRNUF (5’-TGMMGCTGTAYTTGGC-3’) and CRN13R (5’-TTCATCATGAGTGGGTCRTC-3’). The CRNUF primer was designed based on the conserved LxLYLAR/K motif located at the 5’ end of *L. giganteum* CRN genes [6], whereas the CRN13R primer was designed within the DDC motif characteristic of the 3’ end of CRN13 homologs [10]. Polymerase Chain Reaction (PCR) products were purified and sequenced commercially (MacrogenUSA). The resulting sequences were used to design Gene Specific Primers (GSPs) and obtain complete transcript sequences through Rapid Amplification of cDNA Ends (RACE) PCRs, as previously described [6]. Following *in silico* translation, the predicted protein N terminal sequences were scanned for signal peptides using PHOBIUS [36].

### Amplification and sequencing of Glycoside Hydrolase family 20 and family 37 (GH20 and GH37) transcripts

Sequence fragments showing homology to GH20 and GH37 genes were identified as part of an on-going *L. giganteum* transcriptome analysis [6]. Novel next generation sequencing raw sequence reads were obtained from *L. giganteum* ARSEF #373 using the same cDNA library template as described previously [6]. The novel reads obtained for this study were deposited in the NCBI Sequence Read Archive (SRA) database under the accession number SRX2009629 as part of the BioProject PRJNA256125. The GH37 and GH20 sequence reads served as seeds to design GSPs that were used to both confirm the fragments’ sequence information through Sanger-based reactions, and obtain the complete transcript sequences via RACE PCRs, as previously described [6]. The GH20 GSP sequences included HEXF (5’-CATCGTACGCCATCTCACAC-3’) and HEXR (5’-TCGTCATCAATACCGTCGAA-3’). The primers for GH37 included TREHF (5’-TCGGTCTCGGACTACTCTCC-3’) and TREHR (5’-ATCTCCGTCGCGTTGTACTT-3’). Following *in silico* translation, the predicted protein N terminal sequences were scanned for signal peptides using PHOBIUS [36]. Potential for non-classical secretory pathways was tested using SecretomeP v.2.0 [15],using a neural network (NN) output score of 0.5 as previously used for oomycetes [37].

### GH20 phylogenetic analysis

A dataset corresponding to eukaryotic GH20 protein sequences was obtained from a previously published analysis [16]. This dataset was amended with the *L. giganteum* predicted GH20 sequence as well as additional oomycete GH20 protein sequences obtained from FungiDB [38], or from recently published secretomes [5]. In addition, three prokaryotic GH20 protein sequences from the genus *Streptomyces* were downloaded from the NCBI database and added to the dataset to serve as outgroup. Alignments were performed using MUSCLE as embedded in the phylogeny.fr portal [39]. The resulting alignment was inspected visually, validated using the PFAM GH20 Hidden Markov Model (HMM; PF00726), and edited to restrict the analysis to a block ranging from a conserved Proline (P) to a conserved Tryptophan (W) residue (PF00726 HMM positions -2 to 351). The final, aligned dataset consisted of 443 characters for 69 taxa. The best-fit Maximum Likelihood (ML) model for this dataset was identified as LG + I + G by the PROTTEST program [40, 41]. ML analyses that incorporated the model and parameters calculated by PROTTEST were performed using PHYML as embedded in phylogeny.fr. Bootstrap analyses (1000 replicates) and tree editing were also performed using phylogeny.fr [39]. To facilitate comparisons with previous GH20 phylogeny reconstructions, the maximum likelihood analyses were complemented with Bayesian analyses using the WAG amino acid substitution model with a gamma distribution of rate categories and a proportion of invariable sites, as previously described [16]. These Bayesian analyses were performed using MrBayes v.3.2, and were based on 200,000 generations, with a tree sampling frequency of 10 generations, and the exclusion of the first 250 trees to reconstruct the Bayesian consensus tree.

### Differential gene expression analyses in presence of mosquito hosts

Mosquito eggs (*Aedes aegypti*) were obtained from the Florida Medical Entomology Laboratory and allowed to develop to late instar larvae. The larvae were frozen, washed in 95 % ethanol, rinsed with sterile water, and used to supplement Peptone Yeast extract Glucose media (PYG; 25 larvae in 50 mL of PYG). This experimental design was established to mirror recent gene expression studies that demonstrated that autoclaving microbiological media supplemented with insects destroyed insect RNA [42]. Following a 5-7 days growth period in either PYG or PYG supplemented with mosquito larvae, the *L. giganteum* mycelia was processed for RNA extraction as previously described [6]. Total RNA preparations were treated with DNase (Ambion), and the absence of genomic DNA was further validated in RT-PCR reactions using the primers AACSF0 (5’-GGTCGCTGTTTATCATGACG-3’) and AACSR5 (5’-AGACGGTTATCTCCGAAGAGGT-3’), which were designed to flank a conserved oomycete intron located in the 5’ end of the cellulose synthetase 3 gene [24]. The ROCHE Transcriptor and Green Master kits were used for cDNA synthesis and quantitative PCR (qPCR) reactions, respectively. These reactions were performed in a ROCHE LC96 thermocycler using GSPs designed to amplify 100-200 bp fragments of the *L. giganteum* CRN13, GH20 and GH37 genes described in this study, as well as other previously described putative virulence factors, [6]. Four replicates were conducted for each gene. Determination of relative expression ratio for specific gene in the presence vs. absence of the mosquito host was determined from quantitative PCR results in relation to the control genes β-tubulin and cellulose synthetase 3, and performed in the LC96 Application Software [43]. Relative ratio replicates were compared against a ratio of one separately for each gene via independent samples t-tests. All calculations were conducted using an SPSS statistical package (version 19, SPSS inc., IBM). All reported p values are two-tailed with an *a priori* significance level of *p* < 0.05.

## Additional file

Additional file 1: Saprolegnian GH20 sequence alignment. The protein sequences were accessed from GenBank and aligned using MUSCLE. The sequence information that was used to infer the phylogenetic tree presented in Fig. 1 is shaded. These shaded sequences also correspond to the GH20 domain recognized by InterProScan. GenBank accession numbers are shown. (PDF 33 kb)
