# Supplementary material for: Glycoside hydrolases family 20 (GH20) represent putative virulence factors that are shared by animal pathogenic oomycetes, but are absent in phytopathogens
Source: BMC Microbiol. 2016 Oct 6;16:232. doi: 10.1186/s12866-016-0856-7 (PMC5053185; doi:10.1186/s12866-016-0856-7)
Supplement: Additional file 1: — Saprolegnian GH20 sequence alignment. The protein sequences were accessed from GenBank and aligned using MUSCLE. The sequence information that was used to infer the phylogenetic tree presented in Fig. 1 is shaded. These shaded sequences also correspond to the GH20 domain recognized by InterProScan. GenBank accession numbers are shown. (PDF 33 kb) [file 12866_2016_856_MOESM1_ESM.pdf]

```
XP_009833685_Aa       MPEEEPSLGFMTTTTDRTAGGRKETVWVDEPQNSPILSPGGTKFRKGRRICHLVYLVIFL
XP_008874997_Ai       --------------------------------------------------MMIATWPAWL
AIG55611_Tc           ---------------------------------------------------MIWMLVVMM
AIG55828_Ah           ---------------------------------------------------MLARLAA--
XP_012206853_Sp       -----------------------------------------------------------M
XP_008611584_Sd       ---------------------------------------------------MMRILAVAM

XP_009833685_Aa       VIGGITALVIVTTSSSQHSSPPHHKDDELAANVAILKLPVFKYACTENQCVYQNLTKAEV
XP_008874997_Ai       ALSLVSGLVV----------------------------PPRVYQCKDNLCVQEAIA----
AIG55611_Tc           AMAGINGLQV----------------------------PANAYSCVNHRCVAEPRG----
AIG55828_Ah           LIGVAAALQV----------------------------PFTTYECVRGRCEPRPRS----
XP_012206853_Sp       LCSLGAALQV----------------------------PYTTYACENRRCVPQPRT----
XP_008611584_Sd       LSSLGAALQV----------------------------PYTTYACDDRRCVPQPRT----
                        .   .* :                            *   * *    *

XP_009833685_Aa       LSGVPTMGDGLMSLRVCEMTCGNGSMLPMPQ-SIAIASKPGETVAVDVTSFSHHVTTSD-
XP_008874997_Ai       ----FT--ANGTSLSICELTCGDGSLWPKPTKHQDVGRS---VQPISIASITHSVTFLDH
AIG55611_Tc           ----LEVKPGAMGLKLCEMTC-SGNLWPLPW-NVSLAKT---AIAVSIDGIQHDAGFT--
AIG55828_Ah           ----FSPPDSASSLRLCEMTCGAGNLWPLPT-SVSLGTT---TRVVSVDYVSHTVTFLDN
XP_012206853_Sp       ----LTHQASHTSLRLCEMTCGAGNLWPQPT-SSAIAAT---TMAVSVDDVQHSVSFGAG
XP_008611584_Sd       ----LTPQAGHTSLRLCEMTCGAGNLWPQPT-SAAIAAT---TTIVSVDDVQHNVSFGVG
                               .  .* :**:**  *.: * *     :. .   .  :.:  . * .

XP_009833685_Aa       ----SALVKAMQAAFNEHMAAKL-KLAVGGVQDKGASVNVVGTIASAST-----------
XP_008874997_Ai       TNYNSALAPAMQAVFHDVLETKANECTTPDTVPEFFPVSISATITTADE-----------
AIG55611_Tc           ----SSLVHAMQHIFDNTLALKATECVLDAI--NATTLKINATIASFSE-----------
AIG55828_Ah           SVPISPLVGAIQRIFDNTLALKATECALASV--GGAELAVTASIESGNEVRDYFRTFTMA
XP_012206853_Sp       DAVPSHLVRGMQQIFDNALALKATECAFVAV--DSVGLVVDAAIASASE-----------
XP_008611584_Sd       DA--AHLVRSMQHIFDNTLALKATECALVAV--DGIELVVTAAIASASE-----------
                          : *. .:*  * : :  *  : .          : : .:* : .

XP_009833685_Aa       --------ALGLDTDESYEVSI---AATTVTITAKTAFGYRHGLASVVQLVDWCDVSRSF
XP_008874997_Ai       --------RLRVDTNESYTLQVNMTVSPHVSITATTVYGYRHALTTLSQLMEYDELSHTM
AIG55611_Tc           --------ELKLETDESYTLEL---EDGQVLITAATIYGYRHALTTLTQLIEHDDLTHAM
AIG55828_Ah           ADDNTMVQELELETDESYTLTI---VDGAATIHAATVYGYRHALTTLSQLIEYDELSHDM
XP_012206853_Sp       --------TLSLETDESYTLRI---GNGTAVITAATIYGYRHALVTLTQLIEYDELSHAM
XP_008611584_Sd       --------TLSLETDESYTLHI---ANGTASITAATIYGYRHALVTLTQLVEYDELSHTM
                               * ::*:*** : :       . * * * :****.*.:: **::  :::.  :

XP_009833685_Aa       RMVKAVTIQDKPAYKYRGVMLDTARNFHSMAAIKRLVRTMGMHKLNMFHWHITDSSSFPI
XP_008874997_Ai       QIVETASIRDAPAFPHRGITLDTSRNFYSIGAIKRVLDGMGMNKLNTFHWHVTDTNSFPI
AIG55611_Tc           YIITSGIIVDNPFYPHRGVSLDTSRQFYSVASIRRLLDGMGATKLNSFHWHLTDSSSFPI
AIG55828_Ah           HIISAVTITDAPHFAHRGIVLDTSRQYYSVPAIKRLLDGMGATKLNSFHWHFTDTASFPI
XP_012206853_Sp       HMASSASIIDGPAYRHRGVVLDTSRQYYSVAAIRRLLDGMGATKLNTFHWHFTDTASFPV
XP_008611584_Sd       HMASGASIIDGPAYRHRGVVLDTSRQYYSVAAIRRLLDGMGATKLNSFHWHFTDTASFPV
                       :     * * * : :**: ***:*:::*: :*.*::  **  *** ****.**: ***:

XP_009833685_Aa       EIKFEPKFNLYGNYQSDMAYSQANVRDIVAYAKTHGVQVIPEVDAPAHAGAGWQWGPDYD
XP_008874997_Ai       EIIGEPRLTAYGAYSARQVYTQADIRELVQYAKVRGIRVIPELDAPAHVGAGWQWGPDAG
AIG55611_Tc           EIKSEPRLTSNGAFSSHKVYTQREIRELVAYGKARGVRIIPELDAPAHAGAGWQWGAKAG
AIG55828_Ah           EIKGEPRLTAFGAYHPRSVYTQQAMRDIVAYARARGVRVIPEVDAPSHVGAGWQWGKDAG
XP_012206853_Sp       EIKGEPRLTSYGALHPRQIYTQPAIRELVAYAKARGVRVIPEVDAPSHAGAGWQWGVDAG
XP_008611584_Sd       EIKGEPRLTSFGAHHPRQIYTQTAIRGLVAYAKARGVRVIPEVDAPSHAGAGWQWGVDAG
                      **  **.:.  *   .   *:*  :* :* *....*:.:***:***:*.******* . .
```

```
XP_009833685_Aa     VGELTLCWANNPLAYNECYEAPCGQLNPMNEHVYGLLDKVWVEMADMFESDVLHMGGDEV
XP_008874997_Ai     LGDLVVCFDHLPW-QAACVQPPCGQLNPMNPEVYSILDTVYAEFATLFDADVLHMGGDEV
AIG55611_Tc         LGELGVCFQHNPW-PEACVEPPCGQLNPFNDHVYDILDMVYGELDYLFESDVFHMGGDEV
AIG55828_Ah         LGELAVCFGHNPW-TEACVEPPCGQLNPFNPHVYDVLETVYEELNEIFDSDVFHMGGDEV
XP_012206853_Sp     LGELGVCFGHNPW-YDACVEPPCGQLNPFNDHVYDILKTVYSELHGLFDADVFHMGGDEV
XP_008611584_Sd     LGELGVCFGHNPW-YDACVEPPCGQLNPFNDHVYDILKTVYSELHGLFDADVFHMGGDEV
                    :*:* :*: : *     * :.*******:*  **.:*. *: *:  :*::**:*******

XP_009833685_Aa     FTKCWKRSPVVSDAVANTTDDAEYFEISAKFQERVQTNLWKHAPSRKVMLWSSELTSSLY
XP_008874997_Ai     HVGCWNMTDAITQGM-PSRESTDFVNVWGQFQVKAAQLAMAHATNRKIMLWTSDLTHTSV
AIG55611_Tc         HLGCWNMSEAVTKHM-TNRTSKAFYNVWGMFQEKARALV-RQ---EKIAIWTSDLTKAPY
AIG55828_Ah         HLGCWNMSAAVTAHM-TDRSPDAFYRVWGRFQMQARQLV-GE---KKIAVWTSDLTNAPY
XP_012206853_Sp     HLGCWNMTKAVTDHMSSDRSPDAFYHIWGAFQKRARSLVDAH---KKIAIWTSDLTNAPY
XP_008611584_Sd     HLGCWNMTKAITDHMSSDRSPDAFYHIWGAFQTRARSLVDAQ---KKIAIWTSDLTNAPY
                    .  **: : .::  :        :  : . ** ..           *: :*:*:** :

XP_009833685_Aa     -SKYLPKDKIIVHTWAGIQNGNEPRHMADAGYQYVASFQDRHYLDCGHNGIDRKDNGWCA
XP_008874997_Ai     IQRYLPPGQYVIQNWD-ATTTNSTATIAALGYHVVLSNYDKWYLDCGHGNWLTNGTSWCD
AIG55611_Tc         LNKYFDPNSTIVQMWT-LSTSSDAKFYTSQGYSVIASYYDAYYLDCGFGNWILKGSDWCH
AIG55828_Ah         LRKYFDPASTIIQMWT-LSTGSDAARFTAQGYPVIASYYDAYYLDCGFGNWLLKGADWCT
XP_012206853_Sp     LRSYFEPGNTIVQMWT-RSNAGDAAKLTAQGYEVVASYYDAYYLDCGFGNWLRKGDDWCH
XP_008611584_Sd     LRSYFEPSNTIVQMWT-LSNAGDAAKLTAQGYEVIASYYDAYYLDCGFGNWLTKGADWCH
                       *:   . ::: *    . ...   :  **  : *  *  *****...   :. .**

XP_009833685_Aa     PYKTWQVIYEQVLNPNITEDLMPLALGGELVLWTEVSGEASMDVRIWPRAAALAERAWTN
XP_008874997_Ai     PLKSWQVLYDVDMHADLPPELHQFVLGGEAALWSELADEHSAEVKLWPRSAASAERLWTN
AIG55611_Tc         PYHHWSVLYDLNLLHNVPQKHHKLVLGGEVALWSEEADESSMDVKIWPRAAAAAAERWWLN
AIG55828_Ah         PYHHWSVLYDLDVLHNVPAAQRNLVLGGEVALWSEEVDEATMDAKIWPRAAAAAAERWWSN
XP_012206853_Sp     PYHHWSVLYDLDLVQHVPVSQRRLVLGGEVALWSEEVDEATMDAKIWPRAAAAAAERWWTN
XP_008611584_Sd     PYHHWSVLYDLDLVKHVPVAQRRLVLGGEVALWSEEVDEATMDAKIWPRAAAAAAERWWTN
                    * : *.*:*:  :   :.     :.**** .**:*  .* : :..:***:** *** * *

XP_009833685_Aa     PTT--RWDKAMARMTIATYRVVESGSGSDLIQPH--------------------------
XP_008874997_Ai     PST--SWKEAYGRLQRQRNRMMQAGLHADAMQPQ--------------------------
AIG55611_Tc         PLKS-SWKHAIDRIRIHRDRLVDIGIQADAIQPH--------------------------
AIG55828_Ah         PVNG-TWKDAIDRMRIQRDRLVDIGLQADALQPLWCRQNAGDLSQGSGISISATVKSKSE
XP_012206853_Sp     PRNATTWKDAIDRLRIQRDRLVEMGLQADAIQPH--------------------------
XP_008611584_Sd     PRNGTTWKDAIDRLRIQRDRLVEMGLQADAIQPH--------------------------
                    * .   *. *  *:     *::: *  :* :**

XP_009833685_Aa     ------------------------------------------------------------
XP_008874997_Ai     ------------------------------------------------------------
AIG55611_Tc         ------------------------------------------------------------
AIG55828_Ah         ALTVDTDESYELSIDGPKVSINAATVYGYRHALTTLNQLIDYDEISNSVKMIAKAKIADK
XP_012206853_Sp     ------------------------------------------------------------
XP_008611584_Sd     ------------------------------------------------------------

XP_009833685_Aa     ------------------------------------------------------------
XP_008874997_Ai     ------------------------------------------------------------
AIG55611_Tc         ------------------------------------------------------------
AIG55828_Ah         PAYSHRGIVLDTARNYYSIDSLKRLVDTMGANKLNTFHWHFSDSSSFPFEIKSEPRLTSY
XP_012206853_Sp     ------------------------------------------------------------
XP_008611584_Sd     ------------------------------------------------------------
```

```
XP_009833685_Aa      ------------------------------------------------------------
XP_008874997_Ai      ------------------------------------------------------------
AIG55611_Tc          ------------------------------------------------------------
AIG55828_Ah          GAYSKDQVYTQDQIRDFVQFAKARGVRIIPELDAPSHAGAGWQWGPKAGYGELTLCYGSD
XP_012206853_Sp      ------------------------------------------------------------
XP_008611584_Sd      ------------------------------------------------------------

XP_009833685_Aa      ------------------------------------------------------------
XP_008874997_Ai      ------------------------------------------------------------
AIG55611_Tc          ------------------------------------------------------------
AIG55828_Ah          PWMDYCLEPPCGQLNPLNDHVYDILKTVFEEMHGLFDSNVFHMGGDEVSVPCWNSSKVIT
XP_012206853_Sp      ------------------------------------------------------------
XP_008611584_Sd      ------------------------------------------------------------

XP_009833685_Aa      ------------------------------------------------------------
XP_008874997_Ai      ------------------------------------------------------------
AIG55611_Tc          ------------------------------------------------------------
AIG55828_Ah          DHLKNTTSNAPFFDLWGTFQTKAGALIEKANKKIMVWTSDLTTDPYLKYFKPSNTIVQLW
XP_012206853_Sp      ------------------------------------------------------------
XP_008611584_Sd      ------------------------------------------------------------

XP_009833685_Aa      ------------------------------------------------------------
XP_008874997_Ai      ------------------------------------------------------------
AIG55611_Tc          ------------------------------------------------------------
AIG55828_Ah          GGSTDGDAERLTSKGYEVVASYWDAYYLDCGFGGWVSKGNGWCAPYKSWQVIYDLDVRAN
XP_012206853_Sp      ------------------------------------------------------------
XP_008611584_Sd      ------------------------------------------------------------

XP_009833685_Aa      ------------------------------------------------------------
XP_008874997_Ai      ------------------------------------------------------------
AIG55611_Tc          ------------------------------------------------------------
AIG55828_Ah          LTATNAKRVLGSEVAMWSEIADEKAVEAKIWPRAAALAERLWTNPKTNWKSAMTRMRIQR
XP_012206853_Sp      ------------------------------------------------------------
XP_008611584_Sd      ------------------------------------------------------------

XP_009833685_Aa      ----------------WCRQHPGECPLIVWPL
XP_008874997_Ai      ----------------WCQDHPSRCTLL----
AIG55611_Tc          ----------------WCRLNPGECTLL----
AIG55828_Ah          DRIADAGVGTDAVHPLWCRQNPGKCTLV----
XP_012206853_Sp      ----------------WCRLHPGECTLLP---
XP_008611584_Sd      ----------------WCRLHPGECTLLP---
                                     **. :*. *.*:
```
